# The Effect of Job Insecurity and Life Uncertainty on Everyday Consumptions and Broader Life Projects during COVID-19 Pandemic

**DOI:** 10.3390/ijerph18105363

**Published:** 2021-05-18

**Authors:** Antonio Chirumbolo, Antonino Callea, Flavio Urbini

**Affiliations:** 1 Department of Psychology, Sapienza University of Rome, 00185 Rome, Italy; urbiniflavio@gmail.com; 2 Department of Humanities, LUMSA University, 00193 Rome, Italy; a.callea@lumsa.it

**Keywords:** job insecurity, life uncertainty, existential precarity, consumer behaviors, COVID-19 pandemic

## Abstract

Contemporary society is characterized by a high level of uncertainty in many domains of everyday life. The COVID-19 pandemic has generated a deep economic crisis, exacerbating worldwide feelings of uncertainty and precarity. Individuals with insecure jobs have (and will) probably suffered the most from this situation. Workers with higher job insecurity have poorer psychological and physical health, display more negative work attitudes and are less satisfied about their life. However, much less is known about the impact of job insecurity and life uncertainty on consumer behavior. Using the Conservation of Resources theory as a framework, the present study examines a model in which job insecurity and life uncertainty would have a negative effect on everyday consumptions and broader life projects of individuals. Data collection was conducted in Italy in June and July 2020 during COVID-19 pandemic, in the immediate aftermath of the national lockdown. In a sample of 830 workers, the results of a mediation analysis showed that job insecurity and life uncertainty had a detrimental impact of consumer behaviors, since they were significantly associated with higher propensity to sacrifice and reduce everyday short-term consumptions (e.g., buying food) and greater perceived unaffordability of broader long-term life projects (e.g., buying a house).

## 1. Introduction

Over recent years, many scholars have described the labor market as a turbulent environment [1], and the whole of society itself as characterized by chaos [2] and high levels of uncertainty [3,4,5]. In part due to the last global financial crisis of 2008, ever more people are worried about maintaining their jobs. Consequently, many large surveys [6] have pointed out that the perception of job insecurity for several segments of the workforce has increased.

In this regard, the flexible firm model [7] and segmentation theory [8] could provide a theoretical framework to analyze the current situation. In sum, the former splits the workforce within an organization into stable and peripheral groups of employees, whereas the segmentation theory distinguishes between primary and secondary segments in the labor market. Being strongly linked [9], these approaches argue that the primary segment contains the organization’s core group (characterized by higher wage and labor quality) and the secondary segment contains the peripheral group of employees (characterized by lower wage and unfavorable labor quality). From this viewpoint, the current coronavirus (COVID-19) pandemic has pulverized the distinctions between employees’ groups and segments to which they belong, to leave a general sense of uncertainty and insecurity [10], from a social and health point of view [11,12].

Clearly, the COVID-19 pandemic has globally had a negative impact on physical and psychological health, with millions of deaths around the globe and a corresponding increased rate of mental diseases [13,14,15]. Likewise, the economy and workforce have suffered a lot from this situation, although the full economic implications are yet in course of evaluation and are still difficult to estimate so far. With the aim of reducing the transmission of the virus, many countries all over the world have adopted different measures, such as the temporary total lockdown (in Italy, this occurred from 9 March 2020 to 18 May 2020), the closure of non-essential businesses, and the recommendation to work from and stay at home. Consequently, many productive sectors (from large to small enterprises) have been constrained to interrupt their activities, entailing the loss or temporary layoffs for a high number of workers [16].

In this scenario, the COVID-19 pandemic particularly hit the micro-organizations (i.e., with less than ten employees) and those who are self-employed without employees, which together constitute the informal economy. The informal economy refers to all economic activities by workers and economic units that are partially or not covered by formal arrangements [17]. As stated by the International Labour Organization [17] (p. 7), the informal economy “contributes to jobs, incomes and livelihoods, and in many low- and middle-income countries it plays a major economic role”. The informal economy involves about 1.6 of the two billion workers that work in the hardest hit sectors or in small units, such as the accommodation and restaurant sectors, the manufacturing sector, wholesale and retail trade, and agricultural workers, which are all most vulnerable to shocks [17]. The COVID-19 crisis is exacerbating the vulnerabilities and inequalities that already exist in many countries, becoming a possible source of social tension in counties where the informal economy is more widespread [17].

In 2020, ILO [18] estimated an unprecedented global employment loss of 114 million jobs relative to 2019, more severe in terms of losses during the 2008–2009 financial crisis. It is well documented that the COVID-19 pandemic has resulted in large increases in unemployment in many countries [19]. Globally, trends in employment losses vary considerably across the world’s main regions. The losses have been higher in the Americas, and lowest in Europe and Central Asia [20]. Regarding the effects of the COVID-19 pandemic on employment losses across non-metropolitan and metropolitan areas, a substantial difference can be traced. Despite the COVID-19 pandemic being focused in many urban areas, the impacts of employment losses would appear to be greater in large metropolitan areas [21]. The smaller the population in a metropolitan area, the more employment reductions are weakened. Therefore, non-metropolitan areas are generally less vulnerable than metropolitan areas, probably also due to less occasions of virus transmission [22].

With respect to employment status, it is not just workers with standard contracts or those with full-time wage or salary work with a permanent contract who have had an increase in unemployment. As a matter of fact, non-standard contract workers, such as self-employed workers, temporary workers, on-call or part-time contracts and informal economy workers, have also been highly exposed to job and income losses prompted by the COVID-19 pandemic [23].

As the economic situation was worsened by the COVID-19 pandemic, concerns and worries about one’s own employment status have become more and more salient for many employees, with a consequent growth not only of job insecurity perceptions of employees but also of a more general mood of uncertainty expressed by individuals [24].

Evidence appears to point out that, as both unemployment rate and unemployment expectations has increased in 2020, the propensity of households to save has also reached unprecedented levels in response to COVID-19 [25]. Inevitable fallouts on consumer behaviors also include a reduction in private consumption, estimated in 2020 of about minus 30% as compared to 2019 in the EU area [26]. Moreover, these trends appear to extend their influence into 2021, as the European Commission consumer survey suggests that households presume to spend less on most important goods than they did at the beginning of 2020, despite the considerable volume of savings accumulated [26].

Within this general scenario, in the present paper we aimed to investigate the impact of subjective perception of job insecurity on consumer behaviors, considering the role played by the increased general feeling of life uncertainty experienced by individuals during the period of COVID-19 pandemic.

### 1.1. Job Insecurity and Its Outcomes

Although early studies about job insecurity have thirty years of tradition, research on its consequences has exhibited a marked increase since 2000 [27] and the scientific interest toward this topic is still high. Classically, job insecurity has been defined as “perceived powerlessness to maintain desired continuity in a threatened job situation” [28] (p. 438). Several authors provided further definitions of job insecurity, on the one hand referring to the threat or the fear of involuntary job loss [29,30] and on the other hand to concerns about the continued existence or retaining their actual job in the future [31,32].

Although having different definitions, these authors share common elements that characterize job insecurity. First, it concerns a subjective and perceived concern, different to a real risk situation. In fact, different employees who even share the same objective situation (for example, the same contract type in the same organization) may feel different levels of job insecurity [33]. Second, the lack of job continuity is future oriented, but it concerns the present job in the current organization [34]. Third, job insecurity is only related to involuntary job loss, i.e., the definition excludes the case in which employees spontaneously choose to leave or change organization [30]. In sum, the individual, organizational and social costs of job insecurity are imminent and anticipated, although the potential stressful event, i.e., the job loss, is uncertain, future oriented, and involuntary.

Referring to Greenhalgh and Rosenblatt [28], Hellgren and colleagues [35] further identified two different components of job insecurity. The first one concerns the loss of the job itself, labeled quantitative job insecurity. The second refers to perceptions of the potential loss of important aspects of the job, i.e., career opportunities, working conditions, and salary development, and was named qualitative job insecurity. These two dimensions of job insecurity, quantitative and qualitative job insecurity, became by far the dominant typology in job insecurity research and both the fear of job loss and the fear of losing salient qualities of the job have been well recognized as powerful and potential stressor [27,36,37]. In line with this perspective [35] and stress theories [38,39,40], Chirumbolo and colleagues [41] proposed an integrated definition conceiving job insecurity as “a subjective employee’s appraisal of the likelihood and concern for a future involuntary job loss of the current job position and/or for valued job features, based on his/her own evaluation of the work environment” (p. 36).

Several reviews and meta-analyses [1,27,37,42] have widely documented the negative consequences of quantitative job insecurity on health (e.g., worse mental and psychological well-being, general lower well-being, more somatic complaints), job attitudes (e.g., less job satisfaction and commitment), and organizational behaviors (e.g., impaired job performance, less organizational citizenship behavior). The qualitative dimension of job insecurity has received less attention compared to the quantitative one [34,43]. However, in recent years, the scientific interest about the consequences of qualitative job insecurity increased substantially. Empirical evidence has shown that qualitative job insecurity reduces job satisfaction and commitment [44], as well as health and psychological well-being [45]. Furthermore, the threat of losing some valued features of a job negatively impacts on job performance and task performance [43,46]. Overall, findings suggest that the detrimental consequences of qualitative job insecurity are similar, or even greater, to those of quantitative ones, and are deserving of a deeper examination.

### 1.2. Job Insecurity and Consumer Behavior

As previously reviewed, job insecurity research has principally focused on its health, organizational and social consequences. Much less is known about the impact of job insecurity on consumer behaviors. In economic literature, subjective job loss and unemployment expectations were generally linked to a reduction of consumptions [47,48,49], and to an increase in household savings [50], especially in times of recession and greater uncertainty [51]. These trends appeared to be confirmed in recent times of COVID-19 pandemic [25]. A German economic study revealed that higher job insecurity was generally associated with lower saving [52]. However, when perceptions of financial security were controlled for, evidence for precautionary saving behaviors in the presence of job insecurity was found: Households that were somehow anxious about their financial situation were the ones who tended to increase their saving in the light of higher job insecurity [52].

The few previous studies in work psychology were in line with these findings, suggesting that quantitative job insecurity was associated to a reduced propensity in daily consumption and to a holding back on major life decisions [53,54]. In Table 1, a few examples of consumption items and broader life projects negatively affected by job insecurity are presented. This is not surprisingly, though, as subjective expectations of unemployment are reliable signs of the probability of becoming unemployed: In fact, research found that unemployment fear predicts actual future unemployment, above and beyond other observed objective variables [45]. More importantly, high fears of unemployment were found to be associated with significantly lower levels of wage growth [55] and the subjective perception of higher job insecurity was linked to a more tangible wage inequality in a representative sample of Italian workers [56].

Directly or indirectly related to COVID-19, the recent escalation of job insecurity perceptions appears to have a clear link with smoothing in spending and intensified savings, which could be due to forced and/or precautionary consumer decisions [25]. Some explanation of this phenomena could be related to several reasons, such as: (a) the concern about personal finances due to uncertain duration of the pandemic; (b) the actual economic hardship, due to reduced work hours, pay cuts, and job loss; (c) pessimistic and demoralizing media news about the pandemic consequences on economic and organizational crisis, like the increasing of unemployment rate due to the closing of economic activities (such as firms, restaurants, shops), and the firings occurred in several organizations. Although many economic stressors (including job insecurity) were already present before the pandemic, it is undoubtedly true that COVID-19 has exacerbated many of these issues [57]. In fact, it has been shown that the COVID-19 pandemic, besides worsening pre-existing social inequalities, also created new forms of disparities, amplifying the social distribution of economic vulnerability [58].

Lately, it is noteworthy to mention here that most of the empirical studies referred to quantitative job insecurity, while the role played by qualitative job insecurity in reducing consumer spending, or increasing household savings, was almost completely overlooked and remains comparatively understudied.

### 1.3. Work Precarity and Life Uncertainty in the Times of Pandemic

To various extents, the topic of work precarity has involved contributions from different disciplines, such as anthropology [59], and sociology [60,61]. As a matter of fact, the term “precarity” often referred to certain labor market conditions as Western economies were shifting from full time, permanent job positions to more contingent ones, like temporary and part-time positions. However, these more “objective” meanings of precarity often tended to overlook the subjective, affective experience of insecurity which has instead a remarkable impact on employees, as shown by the psychological literature reviewed in the previous section [1,30]. Indeed, subjective job insecurity is certainly connected to work precarity, as occupational precarity entails nonstandard or atypical employment forms generally associated with a lack of security, regularity and stability, statutory rights, material rewards, working-time arrangements, training programs, protection against poverty and the like [62,63,64,65,66].

However, the distinction between feelings of work precarity (i.e., what it is generally called subjective job insecurity) and objective work precarity (i.e., the occupational status within the labor market) does not tell the whole story about feeling precarious and uncertain in contemporary society [4,5]. In fact, some authors underlined how life is generally precarious [67] and that precarity inevitably entails lives that must deal with uncertainty and instability [68]. This matter became particularly salient in contemporary liquid times [5] in which people are more and more required to construct their lives and identities within a context of growing biographical uncertainty [69]. In this perspective, while acknowledging the contamination between existential and economic precarity, many scholars stressed the importance to separate the vulnerability of the general precariousness of living from the precarity of work stemmed from political, legal, and economic regulations [67,70,71,72]. Existential precarity refers to “the general shared vulnerability of life” [71] (p. 219), and to the feeling of being uncertainty about oneself [73]. In this sense, we more precisely define life uncertainty as the individuals’ perceptions and feelings regarding the precariousness, uncertainty, and temporary nature of one’s present and future life.

Speaking about the “incurably fragmented and atomized” world we are living in, characterized by high levels of uncertainty and unpredictability, Bauman [4] (p. 17) remarked a “general mood of precariousness” which closely follows the trend of economic deregulation. Recent survey data collected during COVID-19 pandemic by the European Parliament, and based on representative samples of the various EU countries, appears to endorse and capture this general mood of precariousness and uncertainty that recently wrapped individuals’ lives and that closely followed the pace of the pandemic [24]. In fact, when asked about “what feeling best describe your current emotional status?”, ‘uncertainty’ resulted to be the most common emotional sentiment felt by European citizens, mentioned by 50% of respondents [24]. Feelings of uncertainty remained the most common emotional status all over the year 2020 (three waves), followed by other negative moods such as ‘helplessness’, ‘fear’, ‘anger’ or ‘frustration’. In the words of Gieseck and Rujin [10], “the coronavirus (COVID-19) pandemic has triggered an unprecedented increase in uncertainty. Substantial uncertainty surrounds all aspects of the pandemic: the infectiousness and lethality of the virus; the capability of healthcare systems to adapt to a surge in demand and to develop a medical solution; the duration and effectiveness of containment measures (such as lock downs and social distancing) and their impact on economic activity and employment; the speed of the recovery once containment measures are eased; and the extent to which the pandemic will permanently impact consumption, investment and growth potential” [10] (p. 61).

Surprisingly enough, there is a strong need of empirical quantitative investigations on the antecedents and consequences of life uncertainty and life precarity [74]. Some scholars have investigated the outcomes of life precarity on psychological health, showing that precariousness of life was a significant predictor of both anxiety and depression [75]. The negative consequences of temporary work in personal and family life appeared more evident in women, as women reported significantly higher scores than men on distrust toward the professional future and on anxiety and depression [75]. More recent research found that precariousness of life mediated the relationship between qualitative job insecurity and mental distress variables, such as emotional exhaustion and psychological symptoms [76]. However, despite being a valuable attempt of a new line of research, the main limitation of these studies was represented by the construct definition and the measure used by these authors to assess life precarity, namely the Precariousness of Life Inventory (PLI) [77,78]. In fact, precariousness of life was conceived as “a subjective perception about the negative impact of the contract term on the present and future” [76] (p. 638). In this perspective, regardless of the label, the PLI did not effectively distinguish life precarity (or uncertainty) from precarity originated from work conditions as the items of the inventory, and its three underlying dimensions, were still referred to work elements (the names of these three dimensions being “Indifference to the work”, “Working perspectives” and “Work’s emotional consequences on everyday life”) [77,78]. Therefore, in our opinion, this construct did not represent a genuine assessment of perceptions of life uncertainty the way we defined it, as its measurement tool was not truly free from references to work.

### 1.4. Aim and Hypotheses of the Present Study

In the present paper, we aimed to study the impact of subjective qualitative and quantitative job insecurity on consumer behavior (i.e., short-term spending in goods and long-term life plans and major purchases), considering the role played by the increased general feeling of life uncertainty experienced by individuals during the period of COVID-19 pandemic. In particular, we have previously conceived the notion of life uncertainty as the feelings and cognitions of existential precarity related to the uncertainty and temporary nature of one’s present and future life. More specifically, by life uncertainty we intended the perception that the terms of present life and future life plans are characterized by a sense of precariousness, temporariness, transience, and instability.

**Figure 1:**
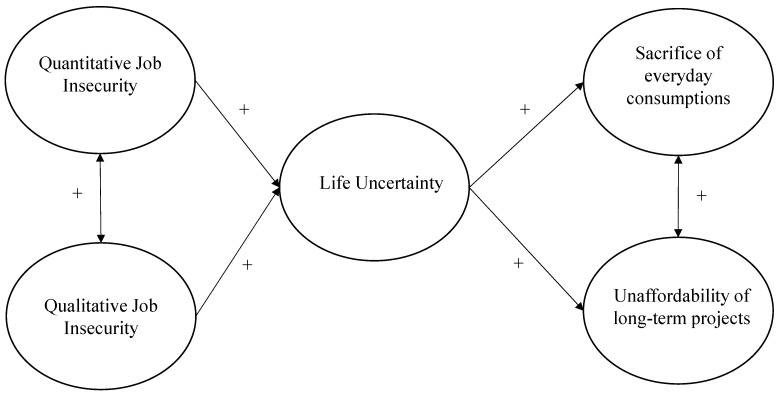
The Theoretical Model.

We hypothesized that higher qualitative and quantitative job insecurity, and higher perceived life uncertainty, were positively related to a higher propensity to sacrifice and reduce everyday short-term consumptions (e.g., buying food, drinks, clothes, entertainments, and the like) and to perceive as unaffordable broader long-term life projects (e.g., getting married, having children, buying a house, obtaining a loan and the like). More importantly, we expected that the perception of life uncertainty mediated the relationship between qualitative and quantitative job insecurity and consumer behavior (see Figure 1 for the theoretical model).

The rationale of the hypotheses can be explained and justified within the theoretical perspective of the Conservation of Resource theory (COR) [79,80,81]. In fact, COR theory can provide a logical explanation about the anticipated effects of job insecurity and life uncertainty on consumer behaviors. As a stress theory, COR theory assumes that threat or actual loss of personal resources may cause strain. In this perspective, perceived job insecurity threatens the resources of both the continuity of present job (job loss i.e., quantitative job insecurity) and of some important features associated to the job itself (loss of salary grown, career development, role, status, i.e., qualitative job insecurity), whereas the perception of precarity and uncertainty of life in general represent an overall personal threat. According to COR, under strains stemmed from facing uncertainty and indefinite circumstances, people tend to cope by adopting a conservative posture and are inclined to preserve their own resources rather than invest into new ones [27]. Therefore, in economic terms, this process would predict that the more insecure employees would tend to save and preserve their own income by reducing their consumption or withholding/postponing some major life decisions which are believed to be less affordable [54].

Moreover, we proposed that life uncertainty would represent an explanatory variable of these relationships. In fact, personal uncertainty represents an aversive and uncomfortable feeling [82,83], if not sometimes even a threatening experience [73]. Therefore, feelings of uncertainty, instability, and precariousness about what may happen in life represent a major stressor and a vulnerability factor [38,39]. We suggested that, on one side, job insecurity would represent an antecedent of perceived life uncertainty. On the other side, life uncertainty would be in turn related to a less proclivity in spending and consuming or make long-term projects.

With respect to previous studies which highlighted a relationship between job insecurity and consumer behavior [47,48,54,84], the present investigation originally contributes to our knowledge in a few ways. Firstly, some of these previous studies have been performed with student participants using fictitious scenarios in experimental design to control feelings of job insecurity [54]. Beside the undoubted advantage to manipulate the independent variable, however, the external validity of such findings may be compromised. Secondly, the subjective perception of objective situation, which represents the core of job insecurity definitions, has not been considered in some other studies that used macro-economic data [48]. Thirdly, previous studies have been only focused on quantitative job insecurity [47,54,84]. However, recent evidence has pointed out the absolute relevance of the consequences of qualitative job insecurity [44,85,86], and therefore empirical proofs of the impact of qualitative job insecurity, and its magnitude, on consumer behaviors are lacking. Lately, we likewise sought to extend findings on the outcomes of life uncertainty on consumer behavior [76]. With the current paper, we aimed to fulfill these gaps present in the literature.

In sum, relying on COR theory [79], and building upon recent studies [84], the present study aimed to investigate in an original way the effects of quantitative and qualitative job insecurity on consumer behaviors, assuming the mediation role of life uncertainty.

## 2. Materials and Methods

### 2.1. Participants and Procedures

The present study was conducted in Italy in June and July 2020 (from June 11th to July 11th), in the immediate aftermath of the national lockdown related to the health emergency. Specifically, Italian workers were web-based recruited through different social media with a snowball sampling procedure and invited to complete an online survey. The link to the survey was sent, posted, and shared using different tools and social networks, e.g., mailing lists, Facebook, WhatsApp, and the like (for similar data collection strategy, see [84,87,88,89,90]).

Overall, 973 questionnaires were gathered. After deleting non-valid questionnaires (due to incompleteness of responses), a total of 830 valid questionnaires were retained resulting in a response rate of 85.5%. The final sample of participants was composed of 830 working adults (M = 261, F = 569, Average age = 47.36 y.o.). The socio-demographic features of participants are fully reported in Table 2.

Before data collection, two different power analyses were run to establish the recommended minimum sample size: (1) for detecting a significant bivariate effect and (2) for conducting a structural equation model (SEM) [91]. We set very conservative parameters in the perspective of a worse-case scenario. A small effect size of *r* = 0.20 was expected, with a power level set at 0.80 and a significant alpha level set at 0.05. The minimum sample size necessary to detect a significant bivariate effect was N = 194 [91]. Regarding the SEM, with the same previous parameters, we considered five latent and fifteen observed variables. Using the software developed by Soper [92], results of this power analysis indicated that the required minimum sample size to run a SEM and detect a significant effect was N = 376, whereas the minimum sample size for model structure was N = 200. Therefore, our sample size appeared more than adequate in terms of statistical power.

The present research was part of a larger project on the impact of job insecurity and was approved the Academic Committee of Sapienza University of Rome (Protocol number RM11816433B7B857).

### 2.2. Measures

The online survey was totally anonymous. In the instructions to the survey, absolute guarantee of anonymity was provided by the researchers. Participants were informed that data would be treated for statistical analyses at an aggregate level and exclusively for scientific purposes. Finally, participants were asked to give their informed consent to participate to the survey by clicking on a button with the following statement: “By clicking Yes, you consent that you are willing to answer the questions in this survey”. The online questionnaire contained a few questions regarding socio-demographic variables and the different measures of our constructs.

Quantitative job insecurity was measured with three items taken from Vander Elst and colleagues [93]. This short scale was intended to assess the perception of being unsecure about one’s own job position. A sample item of this scale is: “I feel insecure about the future of my job”. Participants had to answer on a Likert scale of 5 points, from (1) completely disagree to (5) completely agree. In the present study this measure was unifactorial, with the first factor accounting for 81.39% of the variance (factor loadings ranging from 0.87 to 0.91), and good reliability (Cronbach alpha of 0.88).

Qualitative job insecurity was measured with a scale adapted from Urbanaviciute and colleagues [94] and derived from De Witte and colleagues [95]. It consists of three items measuring perceived negative changes in the overall job content and working conditions. Participants had to answer on a Likert scale of 5 points, from (1) completely disagree to (5) completely agree. A sample item is: “I feel insecure about the characteristics and conditions of my job in the future”. In the present study this measure was unifactorial, with the first factor accounting for 65.83% of the variance (factor loadings ranging from 0.75 to 0.84), and with satisfactory internal consistency (Cronbach alpha of 0.74).

Life uncertainty was measured with a scale of nine items developed ad hoc for the present research. This scale was built to measure the feelings and perceptions of existential precarity, related to the uncertain, precariousness, and provisional nature of one’s present and future life. More precisely, life uncertainty scale assesses the perception that the terms of present life are characterized by a sense of precariousness, temporariness, and instability, and that future life projects are enveloped in an aura of fragility, vagueness, uncertainty, and insecurity. Participants had to answer on a Likert scale of 5 points, from (1) completely disagree to (5) completely agree. Sample items are: “When I think about my situation, I have a feeling that everything is provisional”, “I see my future rather uncertain”. All the items of the scale are exhibited in the Appendix A. In the present study this measure was unifactorial, with the first factor accounting for 66.73% of the variance (factor loadings ranging from 0.68 to 0.87), and with a high internal consistency (Cronbach alpha of 0.94).

Sacrifice of consumptions was measured based on a scale developed by Lozza and colleagues [54,84]. This scale was planned to measure the perceived probability to sacrifice or reduce future spending and consumption of everyday goods, supplies and entertainment. Participants were asked: “How likely do you think you will, in the future, decide to reduce or sacrifice the following categories of consumption?” The following categories were then assessed: (1) High quality food/beverage; (2) Common clothing; (3) Fashion items; (4) Personal care (e.g., creams, make-up, aftershave, perfumes, deodorants etc.); (5) Household tools (e.g., washing machines, microwaves, vacuum cleaners, etc.); (6) Electronics (e.g., smartphones, computers, hi-fi etc.); (7) Cinema, concerts, theater; (8) Holidays (e.g., weekends, summer vacations etc.); (9) Use of private cars and public transport; (10) Restaurants, bars, pubs and pizzerias. Participants had to answer on scale of 5 points scale, from (1) not likely at all to (5) very likely. In the present study this measure was unifactorial, with the first factor accounting for 45.84% of the variance (factor loadings ranging from 0.56 to 0.75), and good reliability (Cronbach alpha of 0.88).

Perceive unaffordability of long-term personal life projects was measured based on a scale developed by Lozza and colleagues [54,84]. Participants were asked: “If you hypothetically intend to, how likely do you think it is that, given your current situation, in the future you can afford to”: (1) Deciding to have children; (2) Rent/Buy/Change home; (3) Buying a car; (4) Log into a mortgage; (5) Get a loan; (6) Getting married. Participants had to answer on scale of 5 points scale, from (1) not likely at all to (5) very likely. In the present study this measure was unifactorial, with the first factor accounting for 58.07% of the variance (factor loadings ranging from 0.70 to 0.85), and reliable (Cronbach alpha of 0.85). Note that this scale was scored so that higher scores meant higher perceived unaffordability.

### 2.3. Data Analyses

Firstly, bivariate correlations among variables were calculated together with descriptive statistics. Secondly, to explore socio-demographic differences in the investigated variables related to gender, age, education, and socio-economic status a series of Multivariate Analyses of Variance (MANOVA) were run. Afterward, a mediation analysis with latent variables was performed via SEM with a partially disaggregated approach [96]. Latent variables of life uncertainty, sacrifice of consumption and unaffordability of life projects were defined using parcels [97,98]. A parcel represents an aggregate of different items measuring a specific construct [97,98]. In the present analysis, three parcels were constructed for each of those latent variables using the item-to-construct balance strategy [98]. Parcels were defined by examining the item-to construct relationships, assessed by the factor loadings at the item-level factor analyses [98]. Usually, parcels that are constructed in this way typically contain a balanced number of items and have a similar reliability. As quantitative and qualitative job insecurity were both assessed by a limited number of items, their corresponding latent variables were defined using items as manifest indicators. Therefore, in the final model, a combination of total and partial disaggregation approach was employed [96].

Model fit was evaluated with different indices: (a) the Comparative Fit Index (CFI); (b) the Tucker–Lewis index (TLI); (c) the root mean squared error of approximation (RMSEA); (d) and the standardized root mean square residual (SRMR). In general, for TLI and CFI, values between 0.90 and 0.95 are considered acceptable [99,100,101] and values above 0.95 are deemed to be very good [102]. On the other hand, RMSEA and SRMR values smaller than (or equal to) 0.08 indicate a good fit [99,101,102,103].

To examine the statistical significance of indirect effects, which represented the “mediated” effects, the bootstrapping procedure was used employing 5000 samples with replacement from the full sample to construct bias corrected 95 percent confidence intervals (CI) [104,105]. Mediation typically occurs if the indirect effect is significant, that is, the zero value is not included in the CI [104,105].

The software SPSS 25 (IBM, Armonk, NY, USA) was used to compute descriptive and correlations, while M-Plus 8.3 was used to test the structural equation model with latent variables and a Maximum Likelihood estimator (ML) was employed to compute all model parameters [106].

## 3. Results

At the bivariate level, both quantitative and qualitative job insecurity was significantly correlated with life uncertainty and sacrifice of consumption. Quantitative job insecurity was also significantly associated with perceived unaffordability of life projects. Life uncertainty was significantly correlated with sacrifice of consumption and perceived unaffordability of life projects. Hypotheses were all supported with the exception that a non-significant correlation between qualitative job insecurity and perceived unaffordability of life projects. Correlations among variables were presented in Table 3, together with descriptive statistics.

### 3.1. Socio-Demographic Differences

In an explorative vein, to investigate possible socio-demographic differences affecting the investigated variables, a series of MANOVAs were run. In these analyses we considered gender, age, education and socio-economic status as factors and quantitative job insecurity, qualitative job insecurity, life uncertainty, sacrifice of consumption, perceived unaffordability of life project as dependent variables. In Table 4, the results of the MANOVA multivariate tests were reported: all effects were significant indicating that, at the multivariate level, the socio-demographic variables affected all the considered dependent variables.

To further interpret these findings, a set of univariate ANOVAs (see Table 5) and descriptive analyses (see Table 6) were also run. As regards gender, females perceived greater unaffordability of life projects with respect to males (Table 5 and Table 6). Regarding age, younger participants reported higher scores on quantitative and qualitative job insecurity, and life uncertainty, while reported lower perceived unaffordability of long-term life projects (Table 5 and Table 6). With respect to education, participants with lower education reported significantly higher scores on quantitative job insecurity, life uncertainty, and sacrifice of daily consumption (Table 4 and Table 5). Finally, participants with a middle-low socio-economic status showed higher scores on quantitative job insecurity, qualitative job insecurity, life uncertainty, sacrifice of consumption, and perceived unaffordability of life projects.

### 3.2. Mediation Model

More importantly, it was hypothesized that life uncertainty would mediate the relationship between qualitative and quantitative job insecurity on one side, and sacrifice of consumption and perceived unaffordability of life projects on the other side (see Figure 1).

In the present paper, we pursued the two-step mediation strategy suggested by [97]. In the first step, the mediation model was tested. This is the hypothesized model without the direct effects from quantitative and qualitative job insecurity to sacrifice of consumption and unaffordability of life projects. This mediation model was indicated with M_med_. In the second step, an unmediated model was tested which included all the direct effects. We indicated this model with M_unmed_. The two nested models were compared via the chi-square difference test, contrasting M_med_ with M_unmed_ (Δχ^2^) [107]. A non-significant Δχ^2^ would mean that the unmediated model did not significantly increase the fit and that, therefore, the mediation model is to be preferred since it is more parsimonious.

**Figure 2:**
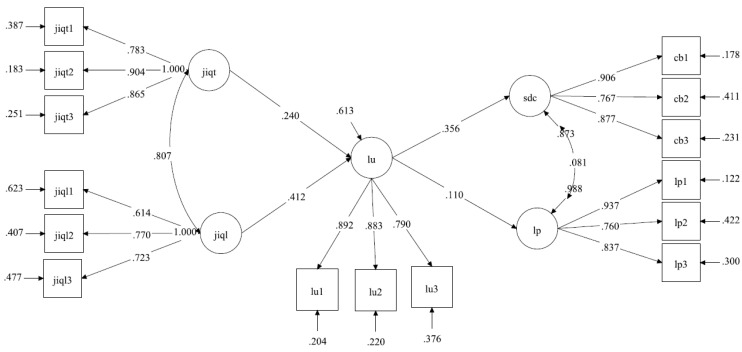
Multivariate Mediation Model with Structural Equation Modeling. Note: Standardized coefficients are reported. All parameters were statistically significant for *p* < 0.05. Jiqt = quantitative job insecurity; jiql = qualitative job insecurity; lu = life uncertainty; sdc = sacrifice of consumption; lp = unaffordability of life projects. Fit Indexes Chi-square (84) = 227.48, *p* < 0.01, CFI = 0.98, TLI = 0.98, RMSEA = 0.04, SRMR = 0.03.

The mediation model (M_med_) showed an overall good fit, chi-square (84) = 227.48, *p* < 0.01, CFI = 0.98, TLI = 0.98, RMSEA = 0.04, SRMR = 0.03. The unmediated model including direct effects (M_unmed_), Chi-square (82) = 225.97, *p* < 0.001; RMSEA = 0.05, CFI = 0.98, TLI = 0.98, SRMR = 0.03, did not significantly improve the model fit. In fact, when the two models were contrasted the resulting chi-square difference was not significant, Δχ^2^ (2) = 1.51, *p* = 0.47. Therefore, the mediation model (M_med_) was preferred because it was more parsimonious in respect to the unmediated model. The parameters of the hypothesized mediation model are reported in Figure 2.

In Table 7, a full decomposition of total and specific indirect effects of the mediated model (M_med_) are reported. All specific indirect effects from both qualitative and quantitative job insecurity to both sacrifice of consumption and unaffordability of life projects, which occurred channeled through life uncertainty, were significant.

When sociodemographic variables such as gender, age, education, and socio-economic status were included in the model as covariates, although slightly worse, the model fit was still satisfactory, Chi-square (136) = 456.81, *p* < 0.01, CFI = 0.96, TLI = 0.95, RMSEA = 0.05, SRMR = 0.05. More importantly for our goals, though, all the relationships among variables (and the indirect effects) were all significant and in the predicted directions.

To rule out likely plausible hypotheses, we also tested an alternative model (M_alt_) in which life uncertainty was the exogenous variable and the two dimensions of job insecurity were the mediators. This alternative model showed an unsatisfactory fit, Chi-square (83) = 507.71, *p* < 0.01, CFI = 0.94, TLI = 0.92, RMSEA = 0.08, SRMR = 0.08. Since the two models M_med_ and M_alt_ were not nested, they could not be compared with the Δχ^2^ test of Satorra and Bentler [108]. Rather, we used the Akaike Information Criterion (AIC) to compare the two models [109]. The AIC of the alternative model (M_alt_) was 31,588.75, while the AIC of the hypothesized mediation model (M_med_) was 31,306.53. Since a lower AIC indicate a better fit, the hypothesized mediation model (M_med_) was to be preferred to the alternative model (M_alt_).

To sum up, the hypothesized mediation model (M_med_) was revealed to be consistent with the data. The unmediated model (M_unmed_) did not fit significantly better than the mediation model (M_med_), and therefore the latter had to be preferred since it was more parsimonious [108]. Direct effects were not significant, while all indirect effects were significant, indicating that a complete mediation actually occurred [104,105,106,107,108]. Additionally, the hypothesized mediation model (M_med_) exhibited a better fit when compared to an alternative model (M_alt_) where both job insecurity dimensions were conceived as mediators.

## 4. Discussion

Contemporary society of the last two decades was generally characterized by high level of uncertainty which have pervaded almost every domain of people’s lives [5]. The recent COVID19 pandemic, though, has undoubtedly exacerbated many of the underlying economic and societal tendencies, dramatically affecting the entire society across the globe. Individuals’ lives suffered of severe consequences in different domains, from psychological health [11,109,110] to employment, finance and business [111,112,113,114], and to consumer behavior as well [115]. As a matter of fact, the feeling of uncertainty has become the most common emotional state experienced by citizens [24]. As the unemployment rate rose, so too did the fear of job loss and future unemployment pairwise increase in the workforce, with consequences on individuals’ financial and consumer behaviors [15,25].

Within this global scenario, the aim of the present paper was to investigate the impact of job insecurity and life uncertainty on consumer behavior during the COVID19 pandemic. Relying on COR theory [79,81], it was predicted that higher feelings of job insecurity and life uncertainty would be related to a reduction in consumption intentions and to a less propensity to undertake major life projects since perceived as less affordable. Facing uncertainty represents a severe strain for most individuals [38]. According to COR theory, when people are under strain and perceive that their resources are being threatened, they tend to assume a more conservative posture and behave consequently to preserve and maintain the threatened resources [79,81]. Undoubtedly, perceived threats to one’s own job position or value aspects of the job (namely qualitative and quantitative job insecurity) represent crucial big risks and pressures for individuals, as their most important resources for living are put in danger. As the same time, perceiving that life and future situations are globally uncertain represents additional threat and strain factors. Under these conditions, we expected that individuals would react in a way to preserve their material resources adopting a saving strategy and a conservative attitude by reducing their short-term spending in goods consumption and sacrificing or postponing major investment and important log-term life projects.

The present findings confirmed our hypotheses, showing that individuals with higher subjective job insecurity and higher life uncertainty reported: (1) a higher probability to sacrifice or reduce their spending in good consumption, such as food, drinks, clothes, entertainments and equipment, and (2) a greater perceived unaffordability, given the actual situation, of hypothetical future major and challenging life investments such as getting married, having children, buying a house, or obtaining a bank loan, the realization of which they will most likely have to renounce or postpone. Moreover, life uncertainty served as an explanatory factor since it mediated the relationships between both qualitative and quantitative job insecurity and consumer behavior.

Dossche and Zlatanos [25] have promptly acknowledged that the propensity of family units to save money has reached unprecedented degrees in response to COVID-19. The authors of [26] outlined at least two major important factors to account for this phenomenon [25]. On one side, the general lockdown measures, which were imposed all over the world to control the spread of the virus, inhibited individuals and families to consuming a great part of their usual expenditure basket, leading to forced and involuntary savings. To put it in other terms, with the closing of shops, pubs, restaurants, cinemas, and the like, some people had fewer opportunities to buy things and spend their money even if they could have done so. People were forced to stay at home and, with many facilities closed, they simply have fewer or even no opportunity to spend their money. This process resulted in a growing tendency to accumulate savings.

On the other side, instead, the sudden outbreak of the pandemic generated uncertainty regarding future income: with the increasing concerns of future unemployment risk, people carried out behaviors of precautionary savings [11]. Precautionary savings mean less propensity to spend in goods and less consumptions. To put in other terms, given the general feeling of insecurity and uncertainty about the future, and given the growing perceived risk of becoming unemployed, some people preferred to spend less and save more money by adopting a cautionary perspective, because “you never know what will happen in the future”. People who had their job at high risk were presumably worried about their earnings and working conditions, but also about their present and future life in general. Therefore, it was very likely for them to take a prudent posture and carry out precautionary savings by reducing their spending and consumptions, and giving up or postpone major investments or life projects since they appear likely unaffordable.

At a macro level, a reduction in consumer and spending behavior was actually observed in times of coronavirus pandemic [115]. If we consider, for instance, the online average monthly spending during the COVID-19 pandemic, compared to the previous year, a substantial reduction in spending was recorded. From a survey conducted by UNCTAD and NetComm Suisse eCommerce Association [116], it can be noted that the greatest decrease in spending was plausibly represented by travel and tourism spending (−75%): “a fact sustained by the multiple flight interdiction, quarantine and lockdowns imposed to tourists traveling from and to different countries” [106] (p. 350). However, many other everyday consumer categories have seen a dramatic reduction as well, from electronic goods (−53%), to fashion (−43%), from cosmetics and personal care (−32%), to education (−29%), from media and books (−27%), to pharmaceutics/health (−23%), to food and beverage (−11%), just to mention a few.

The evidence of the present paper is also in line with previous results reported in scientific literature in a pre-COVID19 era [47,48,49,54,84]. However, we extended prior findings by investigating the impact on consumer behavior of qualitative job insecurity, namely the perception that also value aspects of the job and the general working conditions are been threatened. For instance, a blue-collar or a white-collar employee, or even a self-employed worker, might not have perceived his/her job at risk. However, these workers might have been under pressure as well by perceiving or worrying that, during the pandemic, the general conditions of their job were being threatened and were changing for the worse: for example, getting a lower salary or gaining lower income, receiving less protections and safeguards, being forced to work for more hours with the same income, being under strains derived from working at home (e.g., family-work conflicts), or feeling unable to manage the demands of smart-working conditions, just to mention a few. Precisely, we showed that not only quantitative job insecurity (i.e., fear of losing the job), but also qualitative job insecurity (i.e., fear of worse working conditions) was associated to sacrifice of consumptions. This latter finding was completely overlooked by previous investigations [47,48,49,50,51,52,53,54,84]. Moreover, our results pointed out also the crucial role played by life uncertainty in order to explain the relationship between both job insecurity dimensions and consumer behavior. Again, to our knowledge, no other study has previously investigated the connection between life uncertainty (or existential/life precarity) and consumer behavior.

The present study had some limitations which must be underlined. The first limitation was represented by the cross-sectional design employed in the present investigation. It is well known that such a design does not allow causal inferences. Likewise, we are aware that a cross-sectional design is not the best way to examine mediation effects [117]. Nevertheless, we can draw on previous studies in which the causal link between job insecurity and consumer behavior was verified with experimental designs [54,84] so that we can be confident that the direction would follow our predictions. A second limitation regards the sampling procedure for data collection. The participants were not selected by means of a probabilistic procedure. We used an online snowball sampling procedure which might represent an issue for the lack of representativeness due to selection bias [118]. However, this data collection procedure has been widely employed in current psychological research [84,87,88,89,90]. Moreover, this procedure was the most effective one during COVID-19 pandemic, since traditional paper-and-pencil questionnaires were almost impossible to administer and University laboratories were closed at that time. Future research should verify the generalizability of these results in representative large samples. Nevertheless, the sample was highly heterogeneous and the fact that findings were in line with prior evidence [54,84] and recent analyses at the macro level [25] argues in favor of their robustness.

## 5. Conclusions

The outbreak of the COVID-19 pandemic had severe global consequences, not only in terms of health emergency but also from social, psychological, and economical points of view. A general rise in the general mood of uncertainty and precarity was observed by many analysts. Within this complex scenario, we showed that quantitative and qualitative job insecurity (i.e., the perception and fear of job loss and the loss important job conditions) and life uncertainty (i.e., the perceptions that one’s own life is precarious, instable and uncertain), were significantly associated with a higher propensity to sacrifice purchasing goods of everyday use (e.g., food, drinks, clothes, entertainments) and with the perception of unaffordability of broader long-term life projects (e.g., getting married, have children, buying a house, obtain a loan). Additionally, the findings revealed that life uncertainty mediates the relationship between both job insecurity dimensions and consumer behaviors. Prolonged feelings of uncertainty and insecurity, both at the individual and at a global level, might trigger a vicious circle for the economy that might have potentially harmful and undesirable consequences for people and society at large.

In conclusion, it is worth noting that these findings should be extended and confirmed by conducting longitudinal studies, preferably with several waves and in different countries, in order to measure diachronically the effects of job insecurity and life uncertainty on individuals’ consumer behaviors during COVID-19 pandemic and in its aftermath.

## Figures and Tables

**Table 1:** Example of items about consumer decisions, see also [54].

Everyday Consumptions	Log-Term Life Projects
Housing (e.g., household expenses).	Buying something lasting (e.g., house, car).
Food (e.g., ordinary food, good-quality groceries).	Go living alone.
Clothing (e.g., ordinary and fashionable clothes).	Leave the family of origin.
Beauty care (e.g., cosmetics, personal care products).	Go living together with a partner
Healthcare (e.g., medications, drugs).	Getting married.
Electronic devices (e.g., mobile phones, computers, tablets)	Having children.
Domestic appliances (e.g., fridge).	Renting a house
Leisure activities (e.g., going to theatres, concerts, cinemas, pubs, restaurants, clubs).	Long term economic investments (e.g., obtain a home loan or a bank loan).
Vacations (e.g., weekends, tourism, summer holidays)	Undergo a major surgery.
Use of private vehicles (e.g., car, motorbikes).	Enable a pension plan.

**Table 2:** Individual Characteristics of the Sample (*n* = 830).

Socio-Demographic Characteristics	%
Education level	
1. Middle school	4.2
2. High school	38.3
3. University degree or higher	57.5
Marital status	
1. Single	27.1
2. Married (or lived with a partner)	59.6
3. Divorced	11.3
4. Widowed	1.9
Socio-economic status	
1. Low	5.9
2. Medium low	21.7
3. Medium	59.4
4. Medium high	12.7
5. High	0.04
Contract	
1. Permanent	72.8
2. Temporary	6.5
3. Self employed	12.2
4. No contract	8.6
Occupational status	
1. Full-time	82.7
2. Part-time	6.7
3. Involuntary part-time	3.4
4. Occasional	7.2
Productive sector	
1. Industry	11.1
2. Service	88.0
3. Agricultural	1.0
Organizational sector	
1. Public	63.9
2. Private	36.1
Profession	
1. Blue collars	2.5
2. White collars	76.3
3. Liberal professions	6.9
4. Self-employed	4.9
5. Others	9.3

**Table 3:** Means (M), Standard Deviation (SD), and correlations of study variables.

Variables	M	SD	1	2	3	4	5
1. JIQT	2.02	0.93	1				
2. JIQL	2.36	1.05	0.59 **	1			
3. LU	2.05	0.96	0.52 **	0.49 **	1		
4. SDC	2.49	0.87	0.21 **	0.24 **	0.33 **	1	
5. LP	3.69	1.08	0.09 **	0.05 **	0.12 **	0.12 **	1

Note. ** *p* < 0.01. QTJI = quantitative job insecurity; QLJI = qualitative job insecurity; LU = life uncertainty; SDC = Sacrifice of Consumption; LP = Perceived Unaffordability of Life Project.

**Table 4:** MANOVA’s Multivariate Tests.

	Wilks’ Lambda	F	*p*
Gender	0.97	3.92	0.002
Age	0.90	17.2	<0.001
Education	0.94	10.2	<0.001
Socio-Economic Status	0.96	5.91	<0.001

**Table 5:** MANOVA’s Univariate Tests.

Independent Variables	Dependent Variables	F	*p*
Gender	JI_QT	0.88	0.34
	JI_QL	2.43	0.11
	LU	1.50	0.22
	LP	8.63	0.003
	SDC	2.94	0.08
Age	JI_QT	13.5	<0.001
	JI_QL	4.34	0.03
	LU	14.37	<0.001
	LP	53.41	<0.001
	SDC	1.59	0.20
Education	JI_QT	26.60	<0.001
	JI_QL	0.46	0.49
	LU	16.73	<0.001
	LP	0.075	0.78
	SDC	13.46	<0.001
Socio Economic Status	JI_QT	6.68	0.01
	JI_QL	8.11	0.005
	LU	5.85	0.016
	LP	5.75	0.017
	SDC	22.60	<0.001

Note. QTJI = quantitative job insecurity; QLJI = qualitative job insecurity; LU = life uncertainty; SDC = Sacrifice of Consumption; LP = Perceived Unaffordability of Life Project.

**Table 6:** Means and standard deviations (between brackets) of the studied variables in function of Gender, Age, Education and Socio-Economic Status.

		JI_QT	JI_QL	LU	LP	SDC
Gender	Males	2.07 (0.95)	2.28 (1.07)	2.00 (0.89)	3.53 (1.12)	2.42 (0.84)
	Females	2.00 (0.92)	2.40 (1.04)	2.09 (0.99)	3.77 (1.06)	2.53 (0.89)
Age	<50	2.14 (1.00)	2.44 (1.07)	2.18 (1.02)	3.43 (1.15)	2.53 (0.85)
	≥50	1.90 (0.84)	2.29 (1.03)	1.93 (0.88)	3.96 (0.93)	2.46 (0.89)
Education	University	1.88 (0.85)	2.34 (0.96)	1.94 (0.90)	3.70 (1.04)	2.40 (0.86)
	high school or lower	2.21 (1.01)	2.39 (1.16)	2.22 (1.03)	3.68 (1.15)	2.62 (0.88)
Socio-Economic Status	Middle-High	1.81 (0.83)	2.10 (0.95)	1.85 (0.82)	3.46 (1.23)	2.13 (0.79)
	Middle-Low	2.05 (0.94)	2.40 (1.06)	2.09 (0.98)	3.73 (1.06)	2.55 (0.87)

Note. QTJI = quantitative job insecurity; QLJI = qualitative job insecurity; LU = life uncertainty; SDC = Sacrifice of Consumption; LP = Perceived Unaffordability of Life Project.

**Table 7:** Decomposition of Indirect Effects of the mediated model.

Indirect Effect	Effect	SE	*p*	Bootstrap 95% CI
Indirect Effect: jiqt → lu → sdc	0.085	0.034	0.01	[0.016; 0.132]
Indirect Effect: jiqt → lu → lp	0.026	0.013	0.04	[0.004; 0.062]
Indirect Effect: jiql → lu → sdc	0.147	0.035	0.01	[0.087; 0.237]
Indirect Effect: jiql → lu → lp	0.045	0.019	0.01	[0.018; 0.121]

Note. All effects are standardized coefficients. If the zero-value is not included in the Bootstrap 95% CI, the effect is significant at *p* < 0.05. Jiqt = quantitative job insecurity; jiql = qualitative job insecurity; lu = life uncertainty; sdc: sacrifice of consumption; lp = unaffordability of life projects.

## Data Availability

Data are available under request to the first author.

## Appendix A

**The Life Uncertainty Scale (LUS)**

1. My current life plans are rather vague

2. At the moment, I feel all the fragility of my future plans

3. I see my future rather uncertain

4. My current situation is quite precarious

5. I feel that my life plans are precarious

6. For now, if I think about my life, it seems that everything has a sense of temporari-ness

7. When I think about my situation, I have a feeling that everything is provisional

8. I see my life shrouded in uncertainty

9. My life is surrounded by a feeling of precariousness

## References

[1] Shoss M.K. (2017). Job insecurity: An integrative review and agenda for future research. J. Manag..

[2] Rampini F. (2015). L’età del Caos.

[3] Bauman Z. (2000). Liquid Modernity.

[4] Baumann Z. (2005). Liquid Life.

[5] Baumann Z. (2007). Liquid Times: Living in an Age of Uncertainty.

[6] Eurostat (2015). Quality of Life: Facts and Views.

[7] Atkinson J., Meager N. (1986). New Forms of Work Organization.

[8] Steijn B., Kraan K. (1997). The Labour Market Position of Flexible and Permanent Workers. Reader Flexibilisering van Arbeid en Organisatie.

[9] De Witte H., Näswall K. (2003). ‘Objective’ vs. ‘Subjective’ Job Insecurity: Consequences of Temporary Work for Job Satisfaction and Organizational Commitment in Four European Countries. Econ. Ind. Democr..

[10] Gieseck A., Rujin S. (2020). The impact of the recent spike in uncertainty on economic activity in the euro area. Econ. Bull..

[11] European Central Bank Economic Bulletin 2020, Issue 6, September. https://www.ecb.europa.eu/pub/economic-bulletin/html/eb202006.en.html.

[12] Organization for Economic Co-operation and Development (2020). Strategic Foresight for the COVID-19 Crisis and Beyond: Using Futures Thinking to Design Better Public Policies.

[13] Nearchou F., Flinn C., Niland R., Subramaniam S.S., Hennessy E. (2020). Exploring the impact of CoViD-19 on mental health outcomes in children and adolescents: A systematic review. Int. J. Environ. Res. Public Health.

[14] Vindegaard N., Benros M.E. (2020). COVID-19 pandemic and mental health consequences: Systematic review of the current evidence. Brain Behav. Immun..

[15] Wilson J.M., Lee J., Fitzgerald H.N., Oosterhoff B., Sevi B., Shook N.J. (2020). Job Insecurity and Financial Concern During the COVID-19 Pandemic Are Associated With Worse Mental Health. Occup. Environ. Med..

[16] Bureau of Labor Statistics (2020). The Employment Situation.

[17] International Labour Organization (2021). ILO Monitor: COVID-19 and the World of Work.

[18] International Labour Organization (2021). ILO Monitor: COVID-19 and the World of Work.

[19] Blustein D.L., Duffy R., Ferreira J.A., Cohen-Scali V., Cinamon R.G., Allan B.A. (2020). Unemployment in the time of COVID-19: A research agenda. J. Vocat. Behav..

[20] European Foundation for the Improvement of Living and Working Conditions COVID-19: Implications for Employment and Working Life.

[21] Cho S.J., Lee J.Y., Winters J.V. (2020). Employment Impacts of the COVID-19 Pandemic across Metropolitan Status Size.

[22] Desmet K., Wacziarg R. (2020). Understanding Spatial Variation in COVID-19 across the United States.

[23] International Labour Organization(ILO)-Organisation for Economic Co-operation and Development(OECD) The Impact of the COVID-19 Pandemic on Jobs and Incomes in G20 Economies. ILO-OECD Paper Prepared at the Request of G20 Leaders Saudi Arabia’s G20 Presidency 2020. https://www.ilo.org/wcmsp5/groups/public/---dgreports/---cabinet/documents/publication/wcms_756331.pdf.

[24] Eurobarometer (2020). Public Opinion Monitoring at a Glance in the Time of COVID-19. European Parliament. https://www.europarl.europa.eu/at-your-service/it/be-heard/eurobarometer?year=2020&type=eng.aac.eurobarometer.filters.type.specificSurveys.

[25] Dossche M., Zlatanos S. (2020). COVID-19 and the Increase in Household Savings: Precautionary or Forced?. Econ. Bull..

[26] European Central Bank Economic Bulletin—Statistical Annex 2021, Issue 1, January. https://www.ecb.europa.eu/pub/economic-bulletin/html/index.en.html.

[27] De Witte H., Pienaar J., De Cuyper N. (2016). Review of 30 Years of Longitudinal Studies on the Association Between Job Insecurity and Health and Well-Being: Is There Causal Evidence?. Aust. Psychol..

[28] Greenhalgh L., Rosenblatt Z. (1984). Job insecurity: Toward conceptual clarity. Acad Manage. Rev..

[29] Mohr G.B. (2000). The changing significance of different stressors after the announcement of bankruptcy: A longitudinal investigation with special emphasis on job insecurity. J. Organ. Behav..

[30] Sverke M., Hellgren J., Näswall K. (2002). No security: A meta-analysis and review of job insecurity and its consequences. J. Occup. Health Psychol..

[31] De Witte H. (2005). Job insecurity: Review of the international literature on definitions, prevalence, antecedents and consequences. SA J. Ind. Psychol..

[32] Klandermans B., van Vuuren T. (1999). Job insecurity. Eur. J. Work Organ. Psychol..

[33] Callea A., Urbini F., Ingusci E., Chirumbolo A. (2016). The relationship between contract type and job satisfaction in a mediated moderation model: The role of job insecurity and psychological contract violation. Econ. Ind. Democr..

[34] De Cuyper N., Van Hootegem A., Smet K., Houben E., De Witte H. (2019). All insecure, all good? Job insecurity profiles in relation to career correlates. Int. J. Environ. Res. Public Health.

[35] Hellgren J., Sverke M., Isaksson K. (1999). A two-dimensional approach to job insecurity: Consequences for employee attitudes and well-being. Eur. J. Work Organ. Psychol..

[36] Greenhalgh L., Rosenblatt Z. (2010). Evolution of research on job insecurity. Int. Stud. Manag. Organ..

[37] Sverke M., Hellgren J. (2002). The nature of job insecurity: Understanding employment uncertainty on the brink of a new millennium. Appl Psychol. Int. Rev..

[38] Lazarus R.S., Folkman S. (1984). Stress, Appraisal and Coping.

[39] Lazarus R.S. (1999). Stress and Emotion: A New Synthesis.

[40] Katz D., Kahn R.L. (1978). The Social Psychology of Organizations.

[41] Chirumbolo A., Urbini F., Callea A. (2020). Dimensionality, Reliability and Validity of a Multidimensional Job Insecurity Questionnaire. Preliminary Findings in the Italian Context. Rass. Psicol..

[42] Cheng G.H.L., Chan D.K.S. (2008). Who Suffers More from Job Insecurity? A Meta-Analytic Review. Appl. Psychol. Int. Rev..

[43] Chirumbolo A., Callea A., Urbini F. (2020). Job insecurity and performance in public and private sectors: A moderated mediation model. J. Organ. Eff..

[44] Chirumbolo A., Urbini F., Callea A., Lo Presti A., Talamo A. (2017). Occupations at risk and organizational well-being: An empirical test of a job insecurity integrated model. Front. Psychol..

[45] Callea A., Lo Presti A., Mauno S., Urbini F. (2019). The associations of quantitative/qualitative job insecurity and well-being: The role of self-esteem. Int. J. Stress Manag..

[46] Callea A., Urbini F., Chirumbolo A. (2016). The mediating role of organizational identification in the relationship between qualitative job insecurity, OCB and job performance. J. Manag. Dev..

[47] Benito A. (2006). Does job insecurity affect household consumption?. Oxf. Econ. Pap..

[48] Campos R., Reggio I. (2015). Consumption in the shadow of unemployment. Eur. Econ. Rev..

[49] Stephens M. (2004). Job Loss Expectations, Realizations, and Household Consumption Behavior. Econ. Stat..

[50] Bessho S.I., Tobita E. (2008). Unemployment risk and buffer-stock saving: An empirical investigation in Japan. Jpn. World Econ..

[51] Ravn M.V., Sterk V. (2017). Job uncertainty and deep recessions. J. Monet. Econ..

[52] Klemm M. (2012). Job Security Perceptions and the Saving Behavior of German Households. Ruhr Econ. Paper.

[53] Castiglioni C., Hevierova M., Lozza E. (2019). Changes in job insecurity and extraorganizational outcomes: The effects on consumption and major life decisions in Slovak Republic. Rass. Psicol..

[54] Lozza E., Libreri C., Bosio A.C. (2013). Temporary employment, job insecurity and their extraorganizational outcomes. Econ. Ind. Democr..

[55] Campbell D., Carruth A., Dickerson A., Green F. (2007). Job insecurity and wages. Int. Econ. J..

[56] Scicchitano S., Biagetti M., Chirumbolo A. (2020). More insecure and less paid? The effect of perceived job insecurity on wage distribution. Appl. Econ..

[57] Sinclair R.R., Probst T.M., Watson G.P., Bazzoli A. (2021). Caught between Scylla and Charybdis: How economic stressors and occupational risk factors influence workers’ occupational health reactions to COVID-19. Appl. Psychol..

[58] Qian Y., Fan W. (2020). Who loses income during the COVID-19 outbreak? Evidence from China. Res. Soc. Stratif. Mobil..

[59] Han C. (2018). Precarity, precariousness, and vulnerability. Annu. Rev. Anthropol..

[60] Standing G. (2011). The Precariat: The New Dangerous Class.

[61] Standing G. (2014). A Precariat Charter: From Denizens to CITIZENS.

[62] Kalleberg A.L. (2009). Precarious Work, Insecure Workers: Employment Relations in Transition. Am. Sociol. Rev..

[63] Oddo V.M., Zhuang C.C., Andrea S.B., Eisenberg-Guyot J., Peckham T., Jacoby D., Hajat A. (2020). Changes in precarious employment in the United States: A longitudinal analysis. Scand. J. Work Environ. Health.

[64] Puig-Barrachina V., Vanroelen C., Vives A., Martínez J.M., Muntaner C., Levecque K., Louckx F. (2014). Measuring employment precariousness in the European Working Conditions Survey: The social distribution in Europe. Work.

[65] Reuter M., Wahrendorf M., Di Tecco C., Probst T.M., Chirumbolo A., Ritz-Timme S., Dragano N. (2020). Precarious employment and self-reported experiences of unwanted sexual attention and sexual harassment at work. An analysis of the European Working Conditions Survey. PLoS ONE.

[66] Rodgers G., Rodgers G., Rodgers J. (1989). Precarious work in Western Europe: The state of the debate. Precarious Jobs in Labour Market Regulation: The Growth of Atypical Employment in Western Europe.

[67] Long M. (2015). Precarity, the humanities and slow death. Aust. Health Rev..

[68] Waite L. (2009). A place and space for a critical geography of precarity?. Geogr. Compass.

[69] Leccardi C. (2005). Facing uncertainty: Temporality and biographies in the new century. Young.

[70] Butler J. (2004). Precarious Life: The Powers of Mourning and Violence.

[71] Khosravi S. (2017). Precarious Lives: Waiting and Hope in Iran.

[72] Worth N. (2016). Feeling precarious: Millennial women and work. Environ. Plan. D.

[73] Van den Bos K. (2009). Making sense of life: The existential self trying to deal with personal uncertainty. Psychol. Inq..

[74] Callea A. (2011). Psicologia del Lavoro Atipico.

[75] Callea A., Urbini F., Bucknor D. (2012). Temporary employment in Italy and its consequences on gender. Gend. Manag.

[76] Urbini F., Lo Presti A., Chirumbolo A., Callea A. (2020). Two is worse than one: The mediating role of precariousness of life in the association between qualitative job insecurity and distress among Italian temporary employees. Electron. J. Appl. Stat. Anal..

[77] Callea A. (2010). Un nuovo disagio lavorativo: Validazione del questionario Precarietà di vita [A new working uneasiness: Validation of the precariousness of life inventory]. G. Ital. di Psicol..

[78] Callea A., Urbini F., Lo Presti A. (2016). Valutare la salute dei lavoratori a tempo determinato: Validazione del Precariousness of Life Inventory (PLI-9)—versione breve [Assessing the health of temporary workers: Validation of the Precariousness of Life Inventory (PLI-9)—short version]. Psicol. Della Salut..

[79] Hobfoll S.E. (1989). Conservation of resources: A new attempt at conceptualizing stress. Am. Psychol..

[80] Hobfoll S.E., Folkman S. (2011). Conservation of Resources Theory: Its Implication for Stress, Health, and Resilience. The Oxford Handbook of Stress, Health and Coping.

[81] Hobfoll S.E., Halbesleben J., Neveu J.P., Westman M. (2018). Conservation of resources in the organizational context: The reality of resources and their consequences. Annu. Rev. J. Occup. Organ. Psychol..

[82] Hogg M.A., Zanna M.P. (2007). Uncertainty-Identity Theory. Advances in Experimental Social Psychology.

[83] Van den Bos K., Lind E.A., Zanna M.P. (2002). Uncertainty Management by Means of Fairness Judgments. Advances in Experimental Social Psychology.

[84] Lozza E., Castiglioni C., Bonanomi A. (2020). The effects of changes in job insecurity on daily consumption and major life decisions. Econ. Ind. Democr..

[85] Blotenberg I., Richter A. (2020). Validation of the QJIM: A measure of qualitative job insecurity. Work Stress.

[86] Xiao Z., Wu D., Liao Z. (2018). Job Insecurity and Workplace Deviance: The Moderating Role of Locus of Control. Soc. Behav. Pers..

[87] Bull S.S., Levine D., Schmiege S., Santelli J. (2013). Recruitment and retention of youth for research using social media: Experiences from the Just/Us study. Child. Youth Stud..

[88] Morelli M., Cattelino E., Baiocco R., Trumello C., Babore A., Candelori C., Chirumbolo A. (2020). Parents and Children During the COVID-19 Lockdown: The Influence of Parenting Distress and Parenting Self-Efficacy on Children’s Emotional Well-Being. Front. Psychol..

[89] Rife S.C., Cate K.L., Kosinski M., Stillwell D. (2016). Participant recruitment and data collection through Facebook: The role of personality factors. Int. J. Soc..

[90] Spinelli M., Lionetti F., Pastore M., Fasolo M. (2020). Parents’ stress and children’s psychological problems in families facing the COVID-19 outbreak in Italy. Front. Psychol..

[91] Cohen J. (1988). Statistical Power Analysis for the Behavioral Sciences.

[92] Soper D.S. (2020). Post-hoc Statistical Power Calculator for Hierarchical Multiple Regression. http://www.danielsoper.com/statcalc.

[93] Vander Elst T., De Witte H., De Cuyper N. (2014). The Job Insecurity Scale: A psychometric evaluation across five European countries. Eur. J. Work Organ. Psychol..

[94] Urbanaviciute I., Lazauskaite-Zabielske J., Vander Elst T., De Witte H. (2018). Qualitative job insecurity and turnover intention. Career Dev. Int..

[95] De Witte H., De Cuyper N., Handaja Y., Sverke M., Näswall K., Hellgren J. (2010). Associations between quantitative and qualitative job insecurity and well-being: A test in Belgian banks. Int. Stud. Manag. Organ..

[96] Bagozzi R.P., Heatherton T.F. (1994). A general approach to representing multifaceted personality constructs: Application to state self-esteem. Struct Equ. Modeling.

[97] Coffman D.L., MacCallum R.C. (2005). Using parcels to convert path analysis models into latent variable models. Multivar. Behav. Res..

[98] Little T.D., Cunningham W.A., Shahar G., Widaman K.F. (2002). To parcel or not to parcel: Exploring the question, weighing the merits. Struct Equ. Modeling.

[99] Bollen K.A. (1989). A new incremental fit index for general structural equation models. Sociol. Methods Res..

[100] Byrne B.M. (1994). Structural Equation Modeling with EQS and EQS/Windows: Basic Concepts, Applications and Programming.

[101] Marsh H.W., Hau K.T., Wen Z. (2004). In Search of Golden Rules: Comment on Hypothesis-Testing Approaches to Setting Cutoff Values for Fit Indexes and Dangers in Overgeneralizing Hu and Bentler’s Findings. Struct Equ. Modeling.

[102] Hu L.T., Bentler P.M. (1999). Cutoff Criteria for Fit Indexes in Covariance Structure Analysis: Conventional Criteria Versus New Alternatives. Struct Equ. Modeling.

[103] Browne M.W., Cudeck R., Bollen K.A., Long J.S. (1993). Alternative ways of assessing model fit. Testing Structural Equation Models.

[104] Hayes A.F. (2009). Beyond Baron and Kenny: Statistical Mediation Analysis in the New Millennium. Commun. Monographs.

[105] Preacher K.J., Hayes A.F. (2008). Asymptotic and resampling strategies for assessing and comparing indirect effects in multiple mediator models. Behav. Res. Methods.

[106] Muthén L.K., Muthén B.O. (2017). Mplus User’s Guide.

[107] Satorra A., Bentler P.M. (2001). A scaled difference chi-square test statistic for moment structure analysis. Psychometrika.

[108] James L.R., Mulaik S.A., Brett J.M. (2006). A tale of two methods. Organ. Res. Methods.

[109] Del Rio C., Collins L.F., Malani P. (2020). Long-term health consequences of COVID-19. JAMA.

[110] González-Sanguino C., Ausín B., Castellanos M.Á., Saiz J., López-Gómez A., Ugidos C., Muñoz M. (2020). Mental health consequences during the initial stage of the 2020 Coronavirus pandemic (COVID-19) in Spain. Brain Behav. Immun..

[111] Ali M., Alam N., Rizvi S.A.R. (2020). Coronavirus (COVID-19)—An epidemic or pandemic for financial markets. J. Behav. Exp. Financ..

[112] Donthu N., Gustafsson A. (2020). Effects of COVID-19 on business and research. J. Bus. Res..

[113] Goodell J.W. (2020). COVID-19 and finance: Agendas for future research. Financ. Res. Lett..

[114] Kartseva M.A., Kuznetsova P.O. (2020). The economic consequences of the coronavirus pandemic: Which groups will suffer more in terms of loss of employment and income?. J. Popul. Econ..

[115] Fuciu M. (2020). Effects of the SARS-COV-2 Pandemic on the Marketing and the Consumption Activity. Land Forces Acad. Rev..

[116] UNCTAD and NetComm Suisse eCommerce Association (2020). COVID-19 and E-Commerce, Findings from a Survey of Online Consumers in 9 Countries. https://unctad.org/system/files/official-document/dtlstictinf2020d1_en.pdf108.

[117] Baron R.M., Kenny D.A. (1986). The moderator–mediator variable distinction in social psychological research: Conceptual, strategic, and statistical considerations. J. Pers. Soc. Psychol..

[118] Akaike H. (1987). Factor analysis and AIC. Psychometrika.

